# Sustained Negative BOLD Response in Human fMRI Finger Tapping Task

**DOI:** 10.1371/journal.pone.0023839

**Published:** 2011-08-24

**Authors:** Yadong Liu, Hui Shen, Zongtan Zhou, Dewen Hu

**Affiliations:** College of Mechatronics and Automation, National University of Defense Technology, Changsha, Hunan, People's Republic of China; Beijing Normal University, China

## Abstract

In this work, we investigated the sustained negative blood oxygen level-dependent (BOLD) response (sNBR) using functional magnetic resonance imaging during a finger tapping task. We observed that the sNBR for this task was more extensive than has previously been reported. The cortical regions involved in sNBR are divided into the following three groups: frontal, somatosensory and occipital. By investigating the spatial structure, area, amplitude, and dynamics of the sNBR in comparison with those of its positive BOLD response (PBR) counterpart, we made the following observations. First, among the three groups, the somatosensory group contained the greatest number of activated voxels and the fewest deactivated voxels. In addition, the amplitude of the sNBR in this group was the smallest among the three groups. Second, the onset and peak time of the sNBR are both larger than those of the PBR, whereas the falling edge time of the sNBR is less than that of the PBR. Third, the long distance between most sNBR foci and their corresponding PBR foci makes it unlikely that they share the same blood supply artery. Fourth, the couplings between the sNBR and its PBR counterpart are distinct among different regions and thus should be investigated separately. These findings imply that the origin of most sNBR foci in the finger-tapping task is much more likely to be neuronal activity suppression rather than “blood steal.”

## Introduction

The blood oxygen level-dependent (BOLD) signal is specific to the place and time of metabolic activity [1], [2], [3]. The BOLD signal has therefore been widely used in functional magnetic resonance imaging (fMRI) as a measure of neuronal activity levels in the human cerebral cortex. While much progress has been made in characterizing the hemodynamic response for activations induced by increased metabolic activity, there has been little investigation into the dynamics of deactivation induced by decreased metabolic activity. Many previous studies have reported the existence of a sustained negative BOLD response (sNBR) in visual [4], [5], [6], [7], auditory [8] and somatosensory [9], [10], [11], [12], [13] regions, as well as other brain areas. One study has reported that, when an observer viewed a small, flickering target pattern in a uniform, grey visual field, an extensive area of sNBR could be detected around positive BOLD response (PBR) regions in the primary visual cortex [14]. In 2002, Laurienti and coworkers reported that an ongoing PBR in the visual cortex could be accompanied by an sNBR in auditory cortex, and vice versa. They proposed that these PBR and sNBR counterparts revealed cross-modal neuronal activity [8]. Several human somatosensory fMRI studies have also demonstrated the existence of sNBRs. It has been reported that, when subjects performed a finger-thumb tapping task, sNBRs were present in the ipsilateral primary motor (M1), somatosensory (S1) and subcortical regions [9]. When subjects were asked to perform a right-handed pinch grip repetitively at 1 Hz and at 5% of their individual maximal voluntary contraction, an sNBR was observed in the ipsilateral M1 region. It was concluded that this sNBR mirrored a decrease in cortical excitability [10]. A recent report [11] demonstrated that, when tactile stimuli were delivered to fingers via balloon diaphragms driven by compressed air, a transient NBR was present in ipsilateral rolandic cortex (area 3b of primary somatosensory cortex). This NBR had a shorter duration than did the PBR. Moreover, some voxels in the M1 region exhibited NBRs in response to both ipsilateral and contralateral touch.

It is important to understand the origins of the sNBR and to investigate the coupling between sNBRs and PBRs. These studies may allow for cortical mapping of deactivated neuronal populations and could provide important insights regarding the modulation of attention resources [15]. This work may also reveal the functional and anatomical organization of suppressive or inhibitory circuits throughout the cerebral cortex [7].

Studies examining the sNBR in somatosensory tasks are relatively rare. In this study, we undertook a comprehensive investigation into the differences in spatial structure, area, amplitude, and dynamics between the sNBR and its PBR counterpart while human subjects performed a finger tapping task. Our results suggest that sNBRs are more likely to originate from the suppression of neuronal activity rather than from hemodynamic changes.

## Materials and Methods

### 1. Experiment Design and Data Acquisition

Six right-handed, healthy volunteers (3 males and 3 females, aged 23–30 years) with no history of neurological or psychiatric disorders were recruited from the campus of Chinese Central South University. The present study gained approval from the Ethics Committee of Xiangya Hospital, Chinese Central South University, and written, informed consent was obtained from all subjects.

The task paradigm consisted of five visually cued cycles (each 62.4 seconds in duration) of finger tapping periods (31.2 seconds) alternating with rest periods (31.2 seconds). For the finger tapping task, subjects were instructed to oppose the thumb with the other four fingers. We collected data from the left and right hands separately for each subject. The tapping frequency was approximately 1 Hz. The fMRI data were acquired in a GE Signa system operating at 1.5 T with a gradient echo EPI sequence (TR = 3.12 s, TE = 60 ms, FOV = 24 cm, matrix = 64
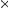
64, slice thickness = 5 mm, gap = 1.5 mm). Eleven oblique slices were acquired with an angle of approximately 20° to the AC-PC plane. These slices were selected to cover the motor representation in the cortex, excluding the cerebellum and the basal ganglia.

### 2. Data Processing

Image sequences were registered to eliminate head movement artifacts. Spatial smoothing was avoided to preserve spatial resolution.

Activated and deactivated voxels were detected by the group spatial independent component analysis (group-sICA) method [16], [17]. This method assumes that, when subjects in an fMRI experiment are carrying out the same task sequence, the underlying hemodynamic response sources from different subjects should exhibit similar dynamics. Only those sources that are included in most or all of the subjects can be separated by this method [16], [17].

The group-sICA model can be expressed as 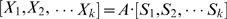. Here, 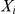 refers to the data matrix of subject *i* with a size of *N* 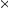 *L_i_*, *N* is the number of scanning points and *L_i_* is the number of voxels inside the brain regions of subject *i*. Accordingly, 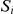 refers to the independent source matrix of subject *i*, *k* is the subject number and the [·] operator denotes a row-wise concatenation of matrices. *A* is the mixing matrix consistent for all subjects. In this study, the fast fixed-point ICA algorithm [18] was applied to estimate *A*. The group image sequence was individually normalized for each subject.

Following group-sICA analysis, the ideal hemodynamic response was correlated with each independent component (IC) separately, and the response with the highest correlation was considered to be task-related. The ideal hemodynamic response was generated by convolving the hemodynamic response function (HRF) with the stimulus function. Here HRF is the one used in SPM software [19]. The task-related component was also inspected manually. For further analysis, the task-related component was transformed into *Z*-score images [20], in which the *Z*-score of a given voxel was computed as the difference between the voxel's task-related contribution and the average task-related contribution across all voxels, divided by the standard deviation of the task-related contribution across all voxels. Voxels with *Z*-score values greater than 5.0 were considered to be activated, while voxels with *Z*-score values less than −5.0 were considered to be deactivated [21]. Here the term ‘activated’ is used to signify that the voxels exhibited PBRs, while the term ‘deactivated’ signifies that the voxels exhibited sNBRs.

We investigated differences in dynamics as well as response amplitudes between a given sNBR and its corresponding PBR. To avoid transient components, we excluded the first block from the analysis, and we averaged the next three blocks for each individual deactivated and activated voxel to generate their mean responses as. In this study, the baseline of a voxel's response was set as its intensity at the scanning point before the onset of the task. For a model-free and comprehensive comparison of time courses, the spline functions were fitted to the mean sNBR and PNR. The fit was established by “cftool” function in Matlab 6.5. The smoothing spline *s* was constructed for the specified smoothing parameter *p*. The smoothing spline minimizes 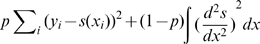, where (*x_i_*, *y_i_*) is the input data and *p* is defined between 0 and 1. *p* = 0 produces a least squares straight line fit to the data, while *p* = 1 produces a cubic spline interpolant. In this work, we choose *p* = 0.5.

## Results

### 1. sNBR exhibits distinct dynamics and amplitudes in different regions

**Figure 1:**
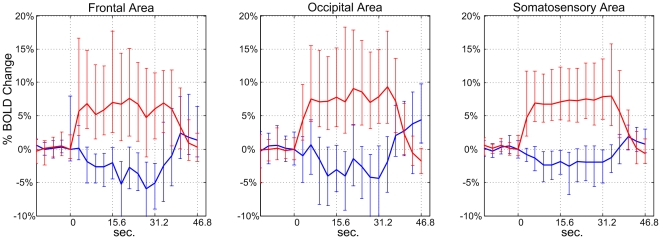
The mean ± s.e.m. of sNBRs and PBRs. The mean ± s.e.m. of sNBRs and PBRs for bilateral frontal, occipital and somatosensory groups taken from left hand data. The first and last blocks of the task paradigm were excluded from analysis, the three middle blocks for each individual deactivated and activated voxel to generate their mean responses, then the SEM were computed over voxels.

**Figure 2:**
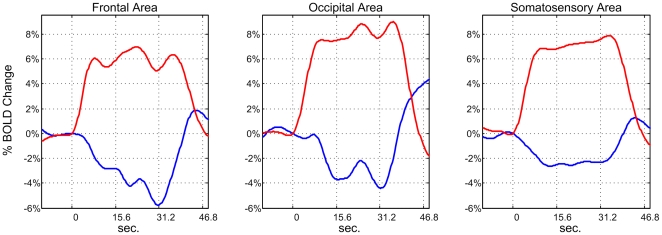
The spline-fitted mean sNBR and PBR. The spline-fitted mean sNBR and PBR values for frontal, occipital and somatosensory groups taken from left hand data.

The dynamics and amplitudes of sNBRs and PBRs were investigated. The activated and deactivated voxels in different hand tasks and different groups were treated separately, and corresponding mean ± standard error of the mean (s.e.m.) values for sNBRs and PBRs were computed. A spline function was fitted to the mean sNBR and PBR for the three groups in both hand tasks. The fitting method is described in the Materials and Methods section. Amplitudes and dynamics of sNBRs and PBRs were measured on spline-fitted responses. We found that sNBRs in different groups exhibited distinct dynamics and amplitudes for both hand tasks. For example, the mean ± s.e.m. responses of the deactivated and activated voxels for the left hand task from the three groups are shown in Figure 1(a), (b) and (c) respectively. Corresponding spline-fitted responses are shown in Figure 2(a), (b) and (c) respectively. To reveal the complete temporal architecture of sNBRs and PBRs, responses in the 12.48 seconds preceding task onset and in the 15.6 seconds following task completion are also shown in the two figures.

In this study, the response amplitude is defined as the maximum/minimum of the percent change for the spline-fitted PBR/sNBR within the [5], [15] scanning point window following the stimulus onset. We found that the amplitude of the sNBR in frontal foci was only slightly smaller than that of its PBR counterpart (5.78%/6.97%≈0.83). This finding is not consistent with previous studies examining somatosensory cortex [9], [11], visual cortex and auditory cortex [6], [8], which reported amplitude ratios between sNBR and PBR of less than 0.5. In contrast, we found that the sNBR/PBR ratios for the occipital and somatosensory foci were 4.40%/8.98%≈0.49 and 2.63%/7.88%≈0.33, respectively; these values are consistent with previous studies [6], [8], [9], [11].

The dynamics of spline-fitted mean sNBR and PBR values were comparatively investigated by measuring their onset time, peak time and falling edge time in the three groups. These dynamic features are listed in Table 1. Here, onset and peak time are respectively defined as the time at which the rising edge of the spline-fitted curve reaches 10% and 90% of its maximum. Falling edge time is defined as the time at which the falling edge reaches 10% of the maximum [22].

**Table 1:** Areas, amplitudes and dynamic features for sNBR and PBR.

	sNBR/PBR	Voxel number	Amplitude (%)	Onset time (Seconds)	Peak time (Seconds)	Falling edge time (Seconds)
**L**	**Frontal foci**	54/36	5.78%/6.97%	3.61/1.64	25.96/15.05	37.80/42.56
**L**	**Occipital foci**	76/58	4.40%/8.98%	3.54/1.86	19.72/17.88	35.83/41.69
**L**	**Somatosensory foci**	44/526	2.63%/7.88%	2.18/1.77	19.17/18.25	36.07/41.80
**R**	**Frontal foci**	38/29	5.12%/5.21%	4.68/1.78	17.85/14.98	37.79/39.12
**R**	**Occipital foci**	76/46	3.65%/6.23%	4.87/1.77	20.56/16.14	37.65/42.31
**R**	**Somatosensory foci**	19/501	2.31%/6.37%	3.76/1.68	19.21/17.24	36.98/42.10

‘R’ denotes right hand data, ‘L’ denotes left hand data.

Compared with the PBR, the sNBR had an increased onset time and peak time but a decreased falling edge time in all three groups. We infer that, in task performance, the sNBR starts later, reaches its peak more slowly and returns to baseline more quickly than does the PBR. When comparing temporal architectures between sNBRs and PBRs, three interesting differences can be found. First, although opposed in sign, the sNBR in the somatosensory and occipital groups presents a similar temporal architecture to its PBR counterpart, which suggests that the sNBR in these two groups is tightly coupled with its PBR counterpart. This further suggests that the mechanisms causing these changes in the BOLD signal in terms of CBF, CBV, and oxygenation may be similar. Second, the sNBR in the frontal group presents a different temporal architecture from its PBR counterpart. Beginning at the onset of the task, the sNBR amplitude continued to increase until the task was finished, indicating that the sNBR is not tightly coupled with its PBR counterpart in the frontal group. Third, for all three groups, the sNBR started to return to baseline within one second of the end of the task, while the PBR maintained a high amplitude for about 5 seconds.

These results suggest that coupling between the sNBR and the PBR is not consistent across cortical regions. Therefore, the sNBR and the PBR should be investigated separately in different regions.

### 2. sNBR is more extensive than that of previously reported

In this study, sNBRs were detected in more brain regions than has previously been reported. According to their clustering, the sNBR foci were classified into frontal, somatosensory and occipital groups. More specifically, the frontal group consisted of the foci in the bilateral superior frontal gyrus and the middle frontal gyrus. The somatosensory group consisted of the foci in the commonly reported ipsilateral somatosensory area, as well as some foci in the contralateral somatosensory area. The occipital group consisted of the foci in the bilateral precuneus, cuneus, superior parietal lobule, and angular gyrus. sNBR foci were not present consistently across all subjects in data from both hands. The locations of sNBR foci for each subject are listed in Table 2, and the numbers of deactivated and activated voxels for the three groups are given in Table 1. In data from both hands, the occipital group contained the most deactivated voxels of the three groups, while the somatosensory group contained the fewest deactivated voxels.

**Table 2:** sNBR foci detected in bilateral hand data.

	Ipsilateral S1	Contralateral S1	Ipsilateral M1	Contralateral M1	Ipsilateral superior frontal gyrus	Contralateral superior frontal gyrus	Ipsilateral middle frontal gyrus	Contralateral middle frontal gyrus
**Subject 1**	L R				L R	L R	L R	L R
**Subject 2**	L			L R	L R	L	L	
**Subject 3**			L R				L R	L R
**Subject 4**	L R	L			L R	L R	R	R
**Subject 5**	L	L			L R	R	L R	
**Subject 6**	L R				L	L	L	R

	Ipsilateral precuneus	Contralateral precuneus	Contralateral cuneus	Ipsilateral cuneus	Ipsilateral superior parietal lobule	Contralateral superior parietal lobule	Ipsilateral angular gyrus	Contralateral angular gyrus
**Subject 1**	R	R		L R	L R	L	L R	L
**Subject 2**	R	L R	L	L R	L R			L R
**Subject 3**	R	R		L	L		L R	L
**Subject 4**	L R	L R	L R	L R	L R	R		R
**Subject 5**								
**Subject 6**	L	L	L R			R	L R	R

‘L’ denotes that this focus exhibits sNBR in left hand data, and ‘R’ denotes that this region exhibits sNBR in right hand data.

We found that the PBR was often present in the bilateral M1 area as well as in the S1 area. In the somatosensory group, the sNBR was often present in the ipsilateral S1 area rather than in the ipsilateral M1 area. Moreover, sNBR areas in S1 exhibited a PBR when subjects performed the contralateral hand motor task, while only a small sNBR was detected in the ipsilateral M1 area. In addition, when tactile stimuli were delivered to three fingers, it has been reported that the ipsilateral S1 area exhibits a transient, negative BOLD response, unlike the sustained response that we have observed [11]. Proprioceptive and tactile feedback are likely to be responsible for the confound between the PBR and sNBR in the bilateral somatosensory region. The simultaneous appearance of a PBR in the contralateral S1 and an sNBR in the ipsilateral S1 could imply that these regions play different roles for opposite hands in motor performance [11].

**Figure 3:**
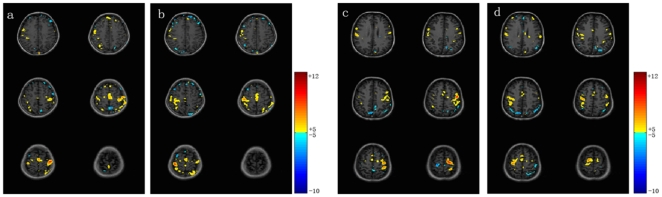
The activation and deactivation maps from two subjects' bilateral hand data. The voxels with *Z*-scores greater than 5.0 are considered to be activated, while the voxels with *Z*-scores less than −5.0 are considered to be deactivated. These voxels are mapped in different colors according to their *Z*-score values. (a) and (b) are for the left and right hand data, respectively, from subject one, while (c) and (d) are for the left and right hand data, respectively, from subject two.

The activation and deactivation maps from two subjects' bilateral hand data are shown in Figure 3. Only the upper six scanning layers, covering most sNBR foci, are shown. The activated and deactivated voxels are mapped in different colors according to their *Z*-score values.

## Discussion

As shown in Table 1, for the somatosensory group, the ratio of the number of deactivated voxels to the number of activated voxels is less than 0.1. Thus, the PBR is the dominant mapping signal in the somatosensory region. In contrast, the ratios for the frontal and occipital groups are both above 1.0, which means that, in these two groups, the sNBR is the primary mapping signal as compared to its PBR counterpart. In most current methods for fMRI analysis (e.g. SPM and sICA), only the PBR is included in the analysis. If the task-specific sNBR does have a neuronal origin, disregarding the sNBR renders it impossible to gain a comprehensive understanding of mechanisms of cortical information processing mechanism. Hence, investigating the origin of the sNBR is of fundamental importance for fMRI studies and other methods in which the sNBR may be involved.

Current interpretations regarding the origin of the sNBR are controversial. Some researchers suggest a hemodynamic origin [23], often regarding the sNBR as a “blood steal” phenomenon. “Blood steal” may redirect blood flow to the activated region and away from adjacent inactive regions [24]. These researchers propose that the sNBR does not reveal the underlying neuronal activity. Others suggest that the sNBR reflects suppression of neuronal activity [5], [7], [9], [11], proposing that the sNBR, like the PBR, can be used as a functional mapping signal.

### 1. The origin of the sNBR observed in this study is largely neuronal activity suppression rather than “blood steal”

We propose that the origin of the sNBR observed in this study is the suppression of neuronal activity rather than “blood steal.” First, if the “blood steal” hypothesis were correct, PBR regions would be expected to “steal” blood from adjacent areas. However, in this study, the distances between sNBRs and adjacent PBR foci are generally too long to share the same blood supply artery. For example, in some data sets, the ipsilateral somatosensory region exhibits an sNBR in the absence of a detectable PBR (for example, the most upper scanning layer in Fig. 3(c)). The left and right hemispheres are fed by different blood supply vessel systems via the two carotid arteries. Blood stealing might occur from nearby capillaries supplied by the same artery, but it is unlikely that blood would be stolen from vessels that are fed by a different artery [15]. Therefore, the sNBR in the ipsilateral somatosensory area cannot be reasonably explained by the presence of “blood steal.” This is also the case for a number of foci in frontal and occipital regions. Second, if the activated voxels need to “steal” blood from certain areas, it is reasonable to suppose that the somatosensory group, which contained the most activated voxels, should require more stolen blood. If this was the case, this region should contain more deactivated voxels than the other two groups. In this study, however, the somatosensory group contained the fewest deactivated voxels of the three groups. In contrast, the frontal and occipital groups had more deactivated voxels than activated voxels. In the context of the “blood steal” hypothesis, it could therefore be inferred that the two less activated regions need comparatively more stolen blood. However, given what is known about the origin of the BOLD response, it is therefore unlikely that the “blood steal” hypothesis is correct. Third, if the “blood steal” hypothesis is correct, the sNBR and the accompanying PBR should present similar temporal architectures. Furthermore, considering that the activated voxels should not steal blood in advance of the task, and considering that blood translation requires some time to occur, the sNBR should exhibit a delay in comparison with the PBR. That is to say, the sNBR should have greater onset, peak and falling edge times than the PBR. However, we did not find this to be the case. First, in frontal regions, the sNBR and PBR exhibited different temporal architectures. Second, in occipital and somatosensory regions, the falling edge time of the sNBR was less than that of the PBR. However, the sNBR and PBR exhibited similar temporal architectures, and the onset and peak time of the sNBR were larger than those of the PBR, This would suggest that the “blood stealing” is completed before the neuronal activity, which is unlikely to be the case.

In this study, we did observe several sNBR foci in frontal, somatosensory and occipital regions that were located near certain PBR regions. These could be explained by the “blood steal” theory. Blood flow in these sNBR foci may be modulated via the dilation of nearby capillaries.

### 2. “Blood sharing” theory

Besides the “blood steal” theory, there is another possibility for the origin of the sNBR. This theory, while it is not popular, is called “blood sharing” [15]. The “blood steal” theory assumes that blood flow in the sNBR areas is regulated via the dilation of the capillary net in PBR areas. However, in the “blood sharing” theory, it is the neuronal population in PBR regions that regulates blood flow in sNBR regions. The “blood sharing” theory assumes that the sNBR does have a neuronal mechanism.

In 1998, it was demonstrated that human cerebral cortex has pericytes around capillaries and smooth muscle cells around arterioles [25]. Via these systems, the neuronal population is able to regulate the blood flow of remote regions, even those in the opposite hemisphere, by controlling the dilation of capillaries and arterioles. However, to date, this neurally-controlled system is not well understood. If this mechanism does exist, it must be a highly developed neural system [25]. However, it is not reasonable to propose that the neuronal population in somatosensory regions need to regulate blood flow in remote areas more strongly than in nearby areas.

### 3. Direct neuronal activity and metabolizable component measurement tools should be exploited

The sNBR may not have a single origin. In some regions or situations, its true origin may be neuronal activity suppression, while the origin may be hemodynamic in other regions or situations. The true origin of the sNBR may be a combination of neuronal and hemodynamic mechanisms. Functional MRI is not powerful enough to distinguish between these possibilities. However, other tools can be exploited to shed light on these mechanisms. These tools include microelectrode arrays [26] and multiwavelength optical imaging [2], [27], which can directly measure ongoing neuronal activity or changes in local metabolizable components in sNBR areas. These tools can reveal whether or not sNBR is tightly coupled with decreases in underlying neuronal activity. For example, an experiment involving simultaneous fMRI and neurophysiological recordings of monkey visual cortex [28] demonstrated that the sNBR is indeed associated with neuronal activity suppression. Couplings between the sNBR and changes in local metabolizable components (e.g., CBV, CBF, and oxygenation) also merit in-depth investigation. Laser Doppler flowmetry or multiwavelength optical imaging techniques may help to shed light on these questions.

### 4. Contribution of this study

This work makes two contributions to the study of NBRs and PBRs. First, the NBRs in the frontal, occipital and somatosensory regions exhibited different temporal architectures than the PBRs. As far as we know, previous studies have not made similar observations. Our finding suggests that the diversity of NBR dynamics should always be considered when using deactivated voxel mapping, hemodynamic identification and anti-correlated network detection. Second, the NBR was detected in more brain regions than has previously been reported. This may be due to our use of group sICA methods in extracting the underlying PBR and NBR foci in this work. In the widely used seed method, the mean time course of seed voxels is selected from some known NBR regions, which in motor tasks is generally the S1 region. This is then correlated with the time course of each voxel. Voxels with a significant correlation are considered to be deactivated. In our study, it was demonstrated that the NBR in frontal regions presents a different temporal architecture than it does in somatosensory and occipital regions. Hence, had the seed method been used to select seed voxels from the S1 region, there is a strong possibility that the deactivated voxels in the frontal region would have been neglected in our analysis. sICA has been demonstrated to be accurate, robust, and successful at detecting voxels with high temporal synchrony [16], [29]. In addition, sICA does not depend on any selected temporal profile of local brain activity; consequently, sICA can successfully extract the NBR in frontal and occipital regions.

### 5. Conclusion

In conclusion, in a finger tapping task, the origin of most sNBRs is likely the suppression of neuronal activity, rather than hemodynamic changes. Like the PBR, the sNBR is an important functional mapping signal in brain imaging. Thus, it is of fundamental importance for fMRI studies to understand the origin of the sNBR and to investigate the coupling between the signal intensity and decreased neuronal activity. To date, studies regarding the origin of the sNBR and its coupling to neuronal activity have not been sufficiently in-depth. Achieving a more comprehensive knowledge of the sNBR requires the use of tools that directly measure neuronal activity and local changes in metabolizable components. These tools include microelectrode arrays and multiwavelength optical imaging. The use of a combination of hemodynamic and neuronal activity measurement tools may provide deep insights into the origin of the sNBR.

## References

[1] Ogawa S, Menton RS, Tank DW (1993). Functional brain mapping by blood oxygen level dependent contrast.. Biophysical Journal.

[2] Boorman L, Kennerley AJ, Johnston D, Jones M, Zheng Y (2010). Negative blood oxygen level dependence in the rat: A model for investigating the role of suppression in neurovascular coupling.. J Neurosci.

[3] Logothetis NK, Brian AW (2004). Interpreting the BOLD signal.. Ann Rev Physiol.

[4] Bressler D, Spotswood N, Whitney D (2007). Negative BOLD fMRI response in the visual cortex carries precise stimulus-specific information.. PLoS ONE.

[5] Tootell R, Mendola JD, Hadjikhani N, Liu AK, Dale A (1998). The representation of the ipsilateral visual field in human cerebral cortex.. Proc Natl Acad Sci USA.

[6] Shmuel A, Augath M, Oeltermann A, Logothetis NK (2006). Negative functional MRI response correlates with decrease in neuronal activity in monkey visual area V1.. Nature NeuroScience.

[7] Pasley BN, Inglis BA, Freeman RD (2007). Analysis of oxygen metabolism implies a neural origin for the negative BOLD response in human visual cortex.. NeuroImage.

[8] Laurienti PJ, Burdette JH, Wallace MT, Yen YF, Field AS (2002). Deactivation of sensory-specific cortex by cross-modal stimuli.. Journal of Cognitive Neuroscience.

[9] Allison JD, Meador KJ, Loring DW, Figueroa RE, Wright JC (2000). Functional MRI cerebral activation and deactivation during finger movement.. Neurology.

[10] Hamzei F, Dettmers C, Rzanny R, Liepert J, Bűchel C (2002). Reduction of excitability (“Inhibition”) in the ipsilateral primary motor cortex is mirrored by fMRI signal decreases.. NeuroImage.

[11] Yevhen H, Riitta H (2006). Transient suppression of ipsilateral primary somatosensory Cortex during Tactile Finger Stimulation.. The Journal of Neuroscience.

[12] Stefanovic B, Warnking JM, Pike GB (2004). Hemodynamic and metabolic responses to neuronal inhibition.. NeuroImage.

[13] Newton JM, Sunderland A, Gowland PA (2005). fMRI signal decreases in ipsilateral primary motor cortex during unilateral hand movements are related to duration and side of movement.. NeuroImage.

[14] Smith AT, Singh KD, Greenlee MW (2000). Attentional suppression of activity in the human visual cortex.. NeuroReport.

[15] Smith AT, Williams AL, Singh KD (2004). Negative BOLD in the visual cortex: evidence against blood stealing.. Human Brain Mapping.

[16] Svensen M, Kruggel F, Benali H (2002). ICA of fMRI group study data.. NeuroImage.

[17] Calhoun VD, Adalv T, Pekar JJ (2004). A method for comparing group fMRI data using independent component analysis: application to visual, motor and visuomotor tasks.. Magnetic Resonance Imaging.

[18] Hyvärinen A, Karhunen J, Oja E (2001).

[19] Friston KJ, Homes AP, Poline JB, Frith C, Frackowiak RSJ (1995). Statistical parametric maps in functional imaging: a general linear approach.. Human Brain Mapping.

[20] McKeown MJ, Jung TP, Makeig S, Brown G, Kinderamann SS (1998). Spatially independent activity patterns in functional MRI data during the Stroop color-naming task.. Proc Natl Acad Sci USA.

[21] McKeown MJ, Makeig S, Brown GG (1998). Analysis of fMRI data by blind separation into independent spatial components.. Human Brain Mapping.

[22] Shmuel A, Yacoub E, Pfeuffer J, Pierre-Francois Van de Moortele, Adriany G (2002). Sustained negative BOLD, blood flow and oxygen consumption response and its coupling to the positive response in the human brain.. Neuron.

[23] Harel N, Lee SP, Nagaoka T, Kim DS, Kim SG (2002). Origin of negative blood oxygenation level-dependent fMRI signals.. Journal of Cerebral Blood Flow Metabolism.

[24] Woolsey TA, Rovainen CM, Cox SB, Henegar MH, Liang GE (1996). Neuronal units linked to microvascular modules in cerebral cortex: response elements for imaging the brain.. Cerebral Cortex.

[25] Rodriguez-Baeza A, Reina de la Torre F, Ortega-Sanchez M, Sahuquillo- Barris J (1998). Perivascular structures in corrosion casts of human central nervous system: a confocal laser and scanning electron microscope study.. Anat Rec.

[26] Yin HB, Liu YD, Li M, Hu DW (2011). Hemodynamic observation and spike recording explain the neuronal deactivation origin of negative response in rat.. Brain Research Bulletin.

[27] Pouratian N, Toga AW (2002). Optical imaging based on intrinsic signals.. Brain Mapping: the Methods(2 edition).

[28] Shmuel A, Augath M, Rounis E, Logothetis NK, Smirnakis S (2003). Negative BOLD response ipsilateral to the visual stimulus: origin is not blood stealing.. NeuroImage.

[29] Van de Ven VG, Formisano E, Prvulovic D, Roeder CH, Linden D (2004). Functional connectivity as revealed by spatial independent component analysis of fMRI measurements during rest.. Human Brain Mapping.

